# Epiretinal Membranes in Patients with Uveitis: Morphological and Functional Analysis with Spectral Domain Optical Coherence Tomography

**DOI:** 10.1155/2013/284821

**Published:** 2013-11-05

**Authors:** Ludovico Iannetti, Paolo Tortorella, Enzo D'Ambrosio, Rossela Spena, Roberta Zito, Magda Gharbiya

**Affiliations:** ^1^Department of Ophthalmology, Sapienza University of Rome, 15500161 Rome, Italy; ^2^Servizio di Immunovirologia Oculare, Sapienza Università di Roma, Viale del Policlinico, 15500161 Rome, Italy

## Abstract

*Purpose*. To correlate the uveitic epiretinal membrane (ERM) features using spectral-domain optical coherence tomography (SD-OCT) with visual acuity (VA). *Methods*. Forty-one eyes of 32 patients were included in this retrospective study. SD-OCT was performed in all patients and data were collected at the time of ERM diagnosis and at the final visit. Both best corrected visual acuity (BCVA) and ERM thickness were correlated with the morphological and clinical features. *Results*. Final BCVA was positively correlated with male sex (*P* = 0.0055) and the focal pattern of ERM attachment (*P* = 0.031) and negatively correlated with IS/OS photoreceptor junction disruption (*P* = 0.042). BVCA change showed a positive correlation with the age of ERM onset (*P* = 0.056) but a negative correlation with IS/OS photoreceptor disruption at the ERM diagnosis (*P* = 0.029) and the increase of central subfield thickness (CST) (*P* = 0.95). Final ERM thickness correlated with the duration of uveitis (*P* = 0.0023) and the duration of ERM (*P* = 1.15  e-05). During the follow-up, ERM thickening correlated with male sex (*P* = 0.042), posterior uveitis (*P* = 0.036), uveitis duration (*P* = 0.026), and broad attachment pattern (*P* = 0.052). *Conclusions*. In the uveitic ERM, VA negatively correlates with IS/OS photoreceptor junction disruption and the increase of CST. ERM thickness is influenced by longer duration of both uveitis and ERM.

## 1. Introduction

Macular epiretinal membrane (ERM) is a pathology caused by a fibrocellular proliferation on the inner limiting membrane (ILM), followed by cellular contraction. ERM can be either idiopathic or secondary to vitreoretinal diseases, such as proliferative vitreoretinopathy (PVR), diabetic retinopathy, and intraocular inflammation. Idiopathic ERM formation is thought to be secondary to glial cell migration, which may require some involvement of retinal pigment epithelial (RPE) cells. On the other hand, the absence of RPE cells and the abundance of inflammatory cells are characteristics of ERM as secondary to uveitis [1].

 Contraction of ERM causes a significant macular dysfunction and is accompanied by the following symptoms: (i) metamorphopsia, (ii) severe visual reduction, and occasionally (iii) central unilateral diplopia [2–4]. Optical coherence tomography (OCT) has become the standard diagnostic technique used to evaluate uveitic macular edema and other pathologies involving the macula in patients with uveitis. The improved resolution and image quality, along with the ease of obtaining these images, have augmented its significance for macular diagnostics in uveitis practice. OCT is suitable for detecting and monitoring uveitic macular edema and provides important information about the fluid distribution in eyes with macular edema as well as revealing the morphology of the vitreoretinal interface. Three different patterns of fluid distribution in the macula of patients with uveitis have been described as follows: cystoid macular edema (CME), diffuse macular edema (DME), and serous retinal detachment. Visual acuity (VA) decreases with increasing fluorescein leakage and central thickness. A correlation has been established between the central thickness measured by OCT and VA. This correlation showed significant differences depending on the OCT pattern and was strongly dependent on the presence of CME. A negative correlation between the central foveal thickness, and VA has also been described in other studies [5–7]. Although DME was associated with poor visual recovery after treatment, some studies indicated a negative visual outcome in patients with uveitic CME [5–7]. The presence of serous retinal detachment in eyes with uveitic macular edema does not appear to worsen visual prognosis. On OCT images, ERM appears as a hyperreflective line adhering to the retina. OCT showed ERM in a higher percentage of cases, compared with those observed ophthalmoscopically in patients with uveitis. In a significant number of eyes with ERM detected ophthalmoscopically, OCT also revealed concurrent vitreoretinal traction; the data confirmed the high sensitivity of OCT in showing the presence of ERM and potential vitreoretinal abnormalities in the macular area. Taken as a whole, this suggests a tractional mechanism as the probable origin or cofactor for the onset of macular edema during uveitis. Studies have shown that ERM is independent of the inflammation site, type of edema, or macular thickness [5–7]. 

ERM can be examined by fundoscopy; however, OCT offers better detection sensitivity [5]. Spectral domain OCT (SD-OCT), the new generation of OCT, was recently introduced into clinical practice. Among the SD-OCT systems, Spectralis OCT (Heidelberg Engineering, Heidelberg, Germany) permits better visualization of the ERM pathologic features and the associated retinal changes [6]. This instrument is equipped with an eye-tracking system that is capable of continuously monitoring the eye position using a light beam, thus increasing the reproducibility of retinal thickness measurements. Cross-sectional B-scans are performed only if the eye-tracking software recognizes the exact location where the image was scanned point-by-point, checking the correlation between the OCT and fundus image [7].

The axial resolution of SD-OCT (generally in the 5–7 *μ*m range for most SD-OCT instruments) is especially useful for a detailed evaluation of the outer retina and for the correlation with visual prognosis. Morphologic SD-OCT parameters have been correlated with visual prognosis in patients involved with idiopathic ERM, but only single studies have reported this correlation in uveitic ERM [8–11]. Nazari et al. demonstrated the negative correlation between inner segment/outer segment (IS/OS) disruption and VA in eyes affected by uveitic ERM; whereas, during a 24-month follow-up central subfield thickness (CST) measurement, the change in the CST compared with the baseline did not correlate with VA. Foveal center involvement, focal attachment of the ERM, and foveal IS/OS junction disruption were each associated independently with lower VA [12, 13]. Additionally, Nazari et al. showed that the central retinal thickness did not correlate with VA; thus, the effect of retinal thickness on VA has yet to be resolved [8, 14–19]. 

The purpose of the present study was to evaluate the characteristics of uveitic ERM using SD-OCT and to correlate the morphological features with VA during the follow-up.

## 2. Materials and Methods

A retrospective study was carried out on all patients with uveitic ERM from July, 2009, to December, 2012. The diagnosis of ERM was performed by clinical examination. Exclusion criteria were as follows: other coexisting ocular diseases limiting VA (e.g., amblyopia, optic atrophy, a macular hole, or central scars). Also, patients who underwent ocular surgery during the observation period (e.g., cataract surgery or a vitrectomy) were excluded. This study was reviewed by the Ethics Committee of The Sapienza University of Rome, and informed consent was obtained from all subjects after the explanation of the nature and possible consequences of the study. The study followed the tenets of the Declaration of Helsinki. The approval of the ethics board allowed children to be included in the study. The collected information included age, sex, anatomic and clinical diagnosis, duration of the uveitis, and clinical examination findings. The Standardization for Uveitis Nomenclature (SUN) Working Group guidelines were used for uveitis anatomic classification and inflammation grading and activity [19]. Best corrected visual acuity (BCVA) was measured.

OCT scans were obtained for each patient using the SD-OCT Spectralis OCT system (Heidelberg Engineering, Germany). Retinal thickness and morphology were assessed by Spectralis OCT, which produces high-resolution cross-sectional B-scans and three-dimensional (3-D) volumetric images, with a speed of up to 40,000 A-scans and a reference fundus image. 

Forty-one eyes of 32 patients with inflammatory ERM (17 males and 15 females), having a median age of 37 years (range: 9–86 years), were included in the study. Spectralis OCT was performed on all patients to identify the fovea and the ERM structural changes during a mean follow-up of 25 ± 11.6 months. Data were collected at the time of ERM diagnosis and at the final visit; the main outcome measures were BCVA, ERM thickness, and CST. Patients with poor-quality SD-OCT images that prevented evaluation and quantification of the SD-OCT data were excluded. An epiretinal thickness map was generated for each subject. The Spectralis OCT imaging protocol used consisted of 37 horizontal B-scans within a 6 × 6 mm scanning area. The boundaries of the ERM were measured manually with calipers provided by the Spectralis OCT system. For manual segmentation, the ERM was defined as the first hyperreflective line internal to the ILM. Segmentation was performed by three independent ophthalmologists. Early Treatment Diabetic Retinopathy Study grid subfields were used to compare the thickness and surface area of the ERM. The central circle, having a 1000 *μ*m diameter centered on the foveal center, was defined as the foveal area; the middle circle (diameter: 3000 *μ*m) was defined as the foveal plus parafoveal area; and the area encompassed in the larger circle (diameter: 6000 *μ*m) was defined as the entire macular area. Spectralis OCT measures retinal thickness as the distance between the ILM and the retinal pigment epithelium base. CST is the mean retinal thickness in the 1000 *μ*m diameter circle centered on the fovea. 

**Figure 1:**
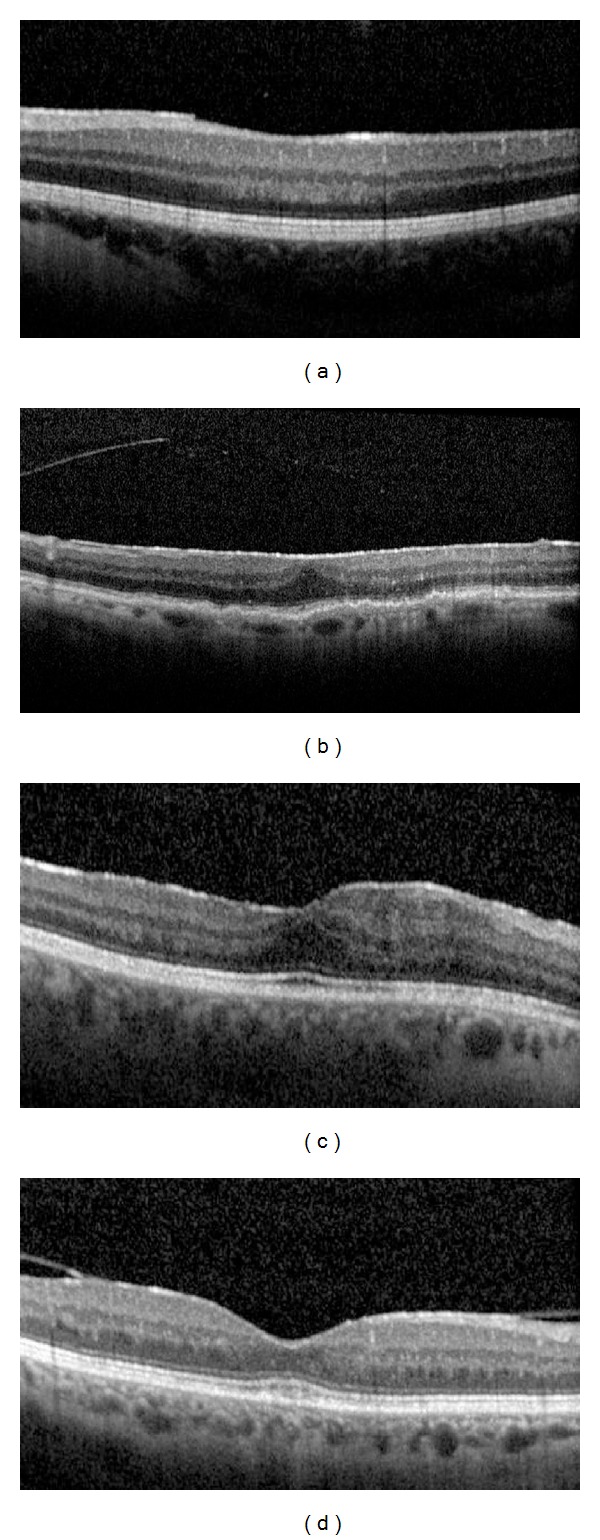
(a) Focal pattern of ERM attachment, (b) broad pattern of ERM attachment, (c) foveal involvement of ERM, and (d) no foveal involvement of ERM.

**Figure 2:**
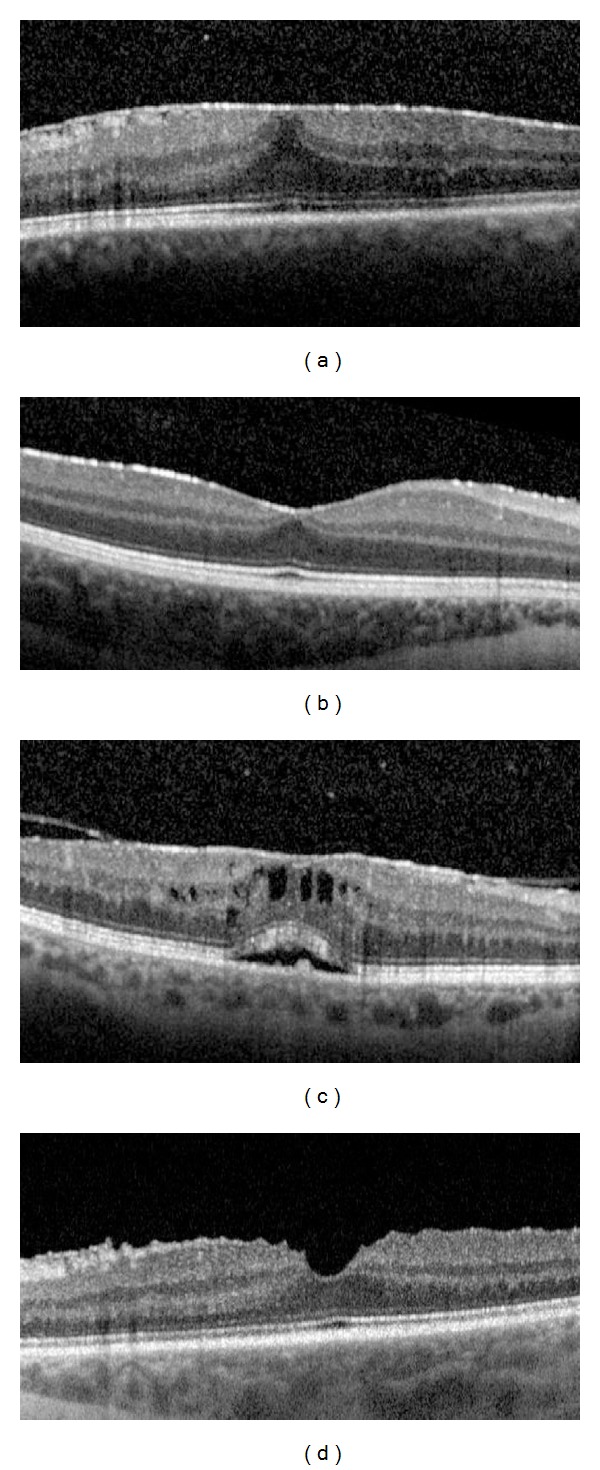
(a) Loss of foveal concavity, (b) presence of foveal concavity, (c) presence of cystoid macular edema, and (d) absence of cystoid macular edema.

**Figure 3:**
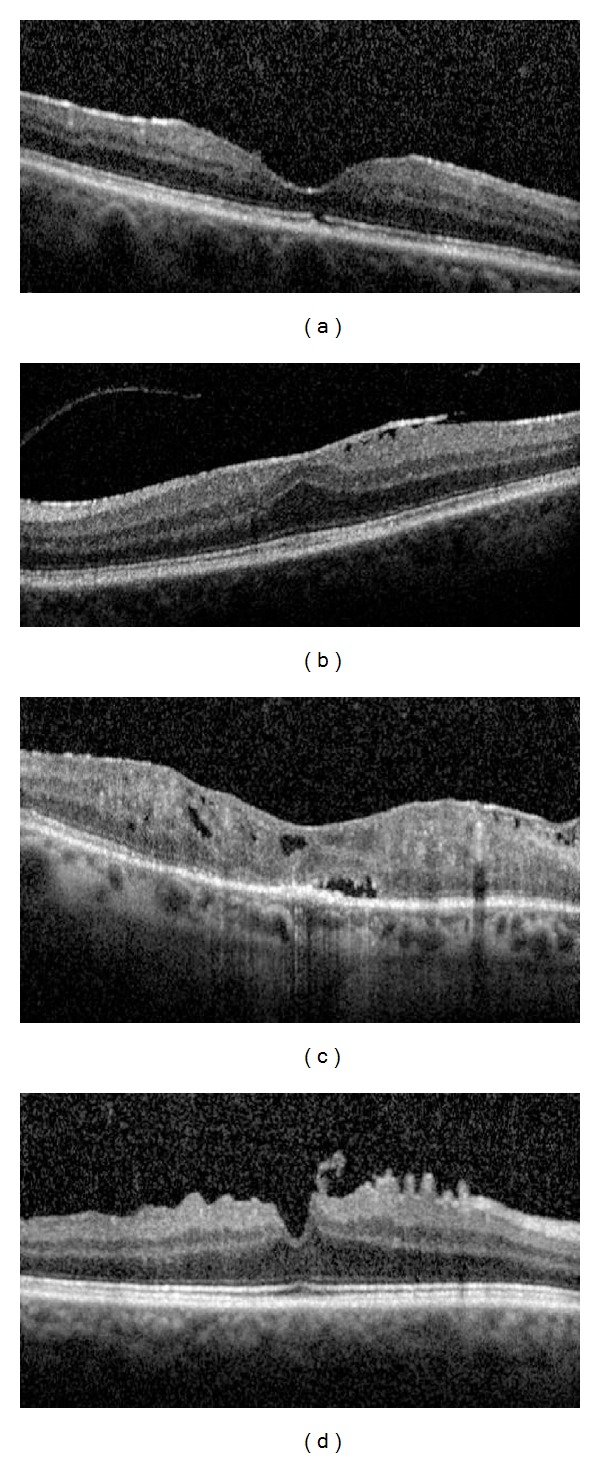
(a) Disruption of IS/OS photoreceptor junction, (b) integrity of IS/OS photoreceptor junction, (c) disruption of ELM, and (d) integrity of ELM.

The correlations between both BCVA and ERM thickness and other parameters, including sex, age, uveitis duration, anatomical site of the inflammation (anterior, intermediate, posterior, and diffuse uveitis), duration of uveitis, duration of ERM, CST, patterns of ERM attachment (Figures 1(a) and 1(b)), foveal involvement (Figures 1(c) and 1(d)), presence or loss of foveal concavity (Figures 2(a) and 2(b)), presence or absence of macular edema (Figures 2(c) and 2(d)), integrity or disruption of photoreceptor IS/OS junction (Figures 3(a) and 3(b)), and the integrity or disruption of the external limiting membrane (Figures 3(c) and 3(d)), were analyzed. The correlation between each of these parameters and the change in the BCVA and ERM thickness was also determined with respect to the first OCT measurement (at the time of ERM diagnosis) and the last OCT measurement (at the last follow-up visit) to study the progression of vision and ERM thickness related to clinical features. All macular B-scans were evaluated for the pattern of attachment (focal or broad) of the ERM to the internal limiting membrane (ILM) of the retina. If both attachment morphologic features were present to a similar extent in different parts of the macula, then the pattern of attachment was recorded as both. 

## 3. Statistical Analysis 

The statistical significance of the change in the ERM thickness and CST from the baseline was assessed using a paired *t*-test. The accepted level of significance corresponded to *P* < 0.05. Multivariate correlation analysis was used to evaluate the BCVA/BCVA change and the ERM thickness/ERM thickness change with respect to the clinical parameters specified above as covariates. 

The decimal BCVA was considered as ordinal data. As seen in the population distribution analysis of BCVA, the underlying population distribution could not be described by a normal distribution, even considering the BCVA above unity with wider gaps between the levels. Moreover, for retrospective studies, the truncation to 10/10 introduces a ceiling effect, making the normality assumption unsuitable (even asymptotically) nor fixable using logMAR conversion. Thus, the median (min-max) value was determined using the Mann-Whitney test. For multivariate analysis, cumulative link modeling (CLM in the ordinal package) was used in the “R for statistical computing” environment, version 2.15.2 [20–22]. Graphical evaluation identified the data outliers, which were eliminated, or used to establish collinearity. For the final BCVA, a flexible threshold with a cloglog link function was suitable. The full model was then evaluated by applying a stepwise procedure in both directions, automatically and manually, to avoid a local optimum. The coefficients for the CLM model did not indicate a correlation coefficient. Thus, the direction and strength of the effect of the covariate were reported graphically, instead of numerically; + (−) was used to represent coefficients between 0 and 1 (−1) and ++ (−−) for coefficients >1 (<−1). *P* values were calculated using the Wald test.

A similar procedure was used in the analysis of the BCVA difference. In this case, the CLM model used an “equidistant” threshold and a cloglog link function. The accepted level of significance was <0.1.

The ERM thickness results were represented by a normal distribution, using the Shapiro-Wilk test. The normality was confirmed for the thickness difference; thus, a generalized linear model (GLM) procedure in R was used. Graphical evaluation identified the outliers or collinearity. The full model was evaluated and optimized through stepwise progression, both automatically and manually. Regression coefficients were reported, along with the *P* values calculated, using a two-tailed *t*-distribution.

## 4. Results

The median decimal BCVA at both the ERM diagnosis and at the final visit was 0.9 (range: 0.02–1.0); the difference was not statistically significant (*P* = 0.9451). The mean initial and final foveal ERM thicknesses were 18.6 ± 6.2 and 22.2 ± 6.0 *μ*m, respectively; this difference was statistically significant (paired *t*-test: *P* = 0.005012). The mean ERM thickness in the foveal plus parafoveal area increased from 19.4 ± 5.1 to 22.9 ± 5.4 *μ*m (paired *t*-test: *P* = 0.00712). The mean initial and final ERM thicknesses of the entire macular area were 19.5 ± 6.3 and 22.7 ± 5.7 *μ*m, respectively (paired *t*-test: *P* = 0.007523). 

 The mean final CST (289 ± 81 *μ*m) was significantly lower than the mean initial CST (312 ± 79 *μ*m) (paired *t*-test: *P* = 0.014). The median duration of uveitis was 10.5 years (range: 2–35 years). The median duration of ERM was 27.5 months (range: 2–42 months). Uveitis was classified according to its inflammation site as follows: 10 anterior, 8 intermediate, 5 posterior, and 9 diffuse. According to the etiological uveitis type, the following was observed: 4 patients with Behçet uveitis, 2 with herpetic uveitis, 3 with Vogt-Koyanagi-Harada (VKH) uveitis, 1 with tuberculosis uveitis (TBC), and 22 with idiopathic uveitis. The clinical features are summarized in Table 1. 

The significant correlations of morphological and clinical features with visual acuity and ERM thickness are summarized in Table 2.

All patients were treated with a combination of topical and systemic corticosteroids and immunomodulatory agents to suppress intraocular inflammation; specifically, 24 patients were treated with systemic steroids, 2 patients with immunosuppressive agents, and 2 patients with biologic agents. During the observation period of the final evaluation, uveitis was determined to be inactive clinically, with respect to baseline measurements. 

## 5. Discussion

Morphological and functional analysis of the inflammatory ERM showed that IS/OS junction disruption and CST were correlated with poor vision; in contrast, the focal pattern of ERM attachment was positively correlated with VA. Visual involvement in eyes with idiopathic ERM may vary from mild to severe, and surgical removal of the ERM in patients with significant visual impairment is commonly performed [23]. However, the visual impact of ERM in eyes with uveitis is not clear. In a recent study, secondary ERMs were associated with worse VA in comparison to idiopathic ERM [24]. Previous studies showed that the alteration of the IS/OS junction is associated with poor VA after idiopathic ERM surgical removal [11, 25, 26]. The layer corresponding to the IS/OS photoreceptor junction appears as a hyper-reflective line under the external limiting membrane. The integrity of this junction during diabetic macular edema is essential for good visual preservation; in contrast, a disruption of the IS/OS junction is correlated with a reduced VA [8, 27]. Oster et al. [8] showed that the disruption of the IS/OS junction is a negative predictor of visual outcome in macular edema secondary to macular pucker. The study of the IS/OS junction integrity appears to be essential for functional evaluation [28].

Our results indicated a negative correlation between BCVA during the final follow-up visit and IS/OS disruption. This was supported by the stronger correlation between the change in BCVA and the presence of IS/OS layer disruption during ERM diagnosis. It is important that this finding is taken into consideration, particularly when planning surgical removal of the ERM. The presence of an intact IS/OS junction on the preoperative SD-OCT images is the most important predictor of better visual recovery and better postoperative BCVA, as indicated in idiopathic ERM [29].

In our analysis, photoreceptor disruption detected by OCT is a predictor of poor visual outcome in eyes with uveitic ERM, and this disruption may be irreversible.

In this series of 32 patients, CST appeared to be associated with a significant decrease in VA by multivariate analysis. This suggests that for these patients, the ERM determining a stronger traction leads to retinal central thickening and consequently a disruption in the IS/OS band. Nazari and Rao observed that during a 24-month follow up, the CST and the change in CST (compared with the baseline) did not correlate with VA in six patients [14]. However, the correlation between CST and final VA remains unresolved [7, 11, 15–18]. 

Our results showed that, in our patients, focal attachment of the membrane was associated with better final vision. This suggests that focal attachment of the ERM, causing traction only in some retinal points, protected most of the retinal tissue from structural changes in the central area. The ERM thickness observed in our patients was associated with ERM duration. Also, the longer duration of uveitis was associated with a thicker ERM. The presence of myofibroblasts, fibroblasts, lymphocytes, and occasional macrophages in the ERMs as well as associated inflammatory cytokines (interferon-*γ*, interleukin-2, interleukin-10, and the tumor necrosis factor) and intravitreal growth factors (TGF*β*2 and NGF) can lead to ERM contraction and its consequent thickening over time [3, 30, 31]. 

Additionally, the posterior localization of uveitis and the broad ERM attachment pattern appeared to influence the ERM thickening, as determined during the follow-up period. Because the uveitis was inactive in the included eyes, the effect of various grades of inflammation and different therapeutic modalities on ERM progression was not studied. A mean follow-up of approximately 2 years following diagnosis indicated that the VA had remained stable, even though significant ERM thickness was evident. Thus, in the group of patients with well-controlled inflammation, even in the presence of ERM thickening, we can expect a good visual outcome. This is in accordance with recent reports that, in most eyes with uveitic ERM, VA remained stable if intraocular inflammation and associated morbidities were treated appropriately. 

## 6. Conclusion

In conclusion, the present study showed that the VA in the uveitic ERM during a long-term follow-up appears to strongly correlate with the following negative prognostic factors: IS/OS photoreceptor junction disruption and the increase of CST. ERM thickness is influenced by longer duration of both uveitis and ERM and seems to be higher in the posterior uveitis and in the ERM with extensive attachment. The analysis of morphological features using SD-OCT is necessary for accurate diagnosis, prognosis, and management of uveitic ERM. During follow-up visits, if the uveitis is well controlled, then an increase in the ERM thickness should not result in vision loss.

## Figures and Tables

**Table 1:** Clinical features of the patients included in the study.

	Initial			Final		
	Nr. of pts.	BCVA median (min–max)	ERM thickness Mean ± sd	Nr. of pts.	BCVA median (min–max)	ERM thickness Mean ± sd
Focal pattern	14	1.0 (0.5–1.0)	19 ± 5	8	1.0 (0.4–1.0)	20 ± 5
Broad pattern	25	0.6 (0.02–1.0)	19 ± 7	31	0.8 (0.02–1.0)	22 ± 6
Both patterns	2	0.95 (0.9–1.0)	16 ± 6	2	0.7 (0.5–0.9)	17 ± 3
Fovea involved	26	0.75 (0.2–1.0)	19 ± 7	32	0.9 (0.1–1.0)	22 ± 6
Fovea not-involved	15	1.0 (0.02–1.0)	19 ± 4	9	1.0 (0.02–1.0)	21 ± 4
Foveal depr present	26	1.0 (0.02–1.0)	18 ± 5	25	1.0 (0.02–1.0)	21 ± 5
Foveal depr absent	15	0.6 (0.2–1.0)	21 ± 7	16	0.6 (0.1–1.0)	23 ± 7
CME present	26	0.7 (0.2–1.0)	18 ± 6	30	0.7 (0.1–1.0)	21 ± 6
CME absent	15	1.0 (0.02–1.0)	20 ± 5	11	1.0 (0.02–1.0)	22 ± 4
IS/OS disruption	3	0.5 (0.2–0.6)	18 ± 7	7	0.5 (0.1–1.0)	23 ± 5
IS/OS integrity	38	1.0 (0.02–1.0)	19 ± 6	34	1.0 (0.02–1.0)	21 ± 6
ELM disruption	1	0.2	10	2	0.45 (0.1–0.8)	27 ± 19
ELM integrity	40	0.9 (0.02–1.0)	19 ± 6	39	0.9 (0.02–1.0)	21 ± 5

**Table:** (a)

Final BCVA		
Covariate	Correlation	*P *
Male sex	++	0.0055
Focal pattern	++	0.031
IS/OS disruption	−	0.042

**Table:** (b)

BCVA change		
Covariate	Correlation	*P *
Age	+	0.056
IS/OS disruption	− −	0.029
CST increase	−	0.095

**Table:** (c)

Final ERM thickness		
Covariate	Corr. Coeff.	*P *
Uveitis duration	0.55	0.0023
ERM duration	0.47	0.000011

**Table:** (d)

ERM thickness change		
Covariate	Corr. Coeff.	*P *
Male Sex	4.50	0.042
Posterior uveitis	6.96	0.036
Uveitis duration	0.26	0.026
Broad ERM pattern	13.6	0.052

## References

[1] Sheybani A, Harocopos GJ, Rao PK (2012). Immunohistochemical study of epiretinal membranes in patients with uveitis. *Journal of Ophthalmic Inflammation and Infection*.

[2] Charles S (2003). Techniques and tools for dissection of epiretinal membranes. *Graefe’s Archive for Clinical and Experimental Ophthalmology*.

[3] Smiddy WE, Maguire AM, Green WR (2005). Idiopathic epiretinal membranes: ultrastructural characteristics and clinicopathologic correlation. *Retina*.

[4] Kampik A, Kenyon KR, Michels RG, Green WR, de la Cruz ZC (2005). Epiretinal and vitreous membranes: comparative study of 56 cases. *Retina*.

[5] Puliafito CA, Hee MR, Lin CP (1995). Imaging of macular diseases with optical coherence tomography. *Ophthalmology*.

[6] Wolf S, Wolf-Schnurrbusch U (2010). Spectral-domain optical coherence tomography use in macular diseases: a review. *Ophthalmologica*.

[7] Iannetti L, Spinucci G, Abbouda A, De Geronimo D, Tortorella P, Accorinti M (2012). Spectral-domain optical coherence tomography in uveitic macular edema: morphological features and prognostic factors. *Ophthalmologica*.

[8] Oster SF, Mojana F, Brar M, Yuson RMS, Cheng L, Freeman WR (2010). Disruption of the photoreceptor inner segment/outer segment layer on spectral domain-optical coherence tomography is a predictor of poor visual acuity in patients with epiretinal membranes. *Retina*.

[9] Arichika S, Hangai M, Yoshimura N (2010). Correlation between thickening of the inner and outer retina and visual acuity in patients with epiretinal membrane. *Retina*.

[10] Legarreta JE, Gregori G, Knighton RW, Punjabi OS, Lalwani GA, Puliafito CA (2008). Three dimensional spectral-domain optical coherence tomography images of the retina in the presence of epiretinal membranes. *American Journal of Ophthalmology*.

[11] Falkner-Radler CI, Glittenberg C, Hagen S, Benesch T, Binder S (2010). Spectral-domain optical coherence tomography for monitoring epiretinal membrane surgery. *Ophthalmology*.

[12] Nazari H, Dustin L, Heussen FM, Sadda S, Rao NA (2012). Morphometric spectral-domain optical coherence tomography features of epiretinal membrane correlate with visual acuity in patients with uveitis. *American Journal of Ophthalmology*.

[13] Unoki N, Nishijima K, Kita M, Hayashi R, Yoshimura N (2010). Structural changes of fovea during remission of Behçet’s disease as imaged by spectral domain optical coherence tomography. *Eye*.

[14] Nazari H, Rao N (2012). Longitudinal morphometric analysis of epiretinal membrane in patients with uveitis. *Ocular Immunology and Inflammation*.

[15] Wilkins JR, Puliafito CA, Hee MR (1996). Characterization of epiretinal membranes using optical coherence tomography. *Ophthalmology*.

[16] Massin P, Allouch C, Haouchine B (2000). Optical coherence tomography of idiopathic macular epiretinal membranes before and after surgery. *American Journal of Ophthalmology*.

[17] Suzuki T, Terasaki H, Niwa T, Mori M, Kondo M, Miyake Y (2003). Optical coherence tomography and focal macular electroretinogram in eyes with epiretinal membrane and macular pseudohole. *American Journal of Ophthalmology*.

[18] Koo HC, Rhim WI, Lee EK (2012). Morphologic and functional association of retinal layers beneath the epiretinal membrane with spectral-domain optical coherence tomography in eyes without photoreceptor abnormality. *Graefe’s Archive for Clinical and Experimental Ophthalmology*.

[19] Jabs DA, Nussenblatt RB, Rosenbaum JT (2005). Standardization of uveitis nomenclature for reporting clinical data. Results of the first international workshop. *American Journal of Ophthalmology*.

[20] R Core Team R: A language and environment for statistical computing. R Foundation for Statistical Computing. http://www.R-project.org/.

[21] Christensen RHB Ordinal-regression models for ordinal data.

[22] Christensen RHB Analysis of ordinal data with cumulative link models-estimation with the R-package ordinal.

[23] Jabs DA (2005). Improving the reporting of clinical case series. *American Journal of Ophthalmology*.

[24] Gupta P, Sadun AA, Sebag J (2008). Multifocal retinal contraction in macular pucker analyzed by combined optical coherence tomography/scanning laser ophthalmoscopy. *Retina*.

[25] Hiscott PS, Unger WG, Grierson I, McLeod D (1988). The role of inflammation in the development of epiretinal membranes. *Current Eye Research*.

[26] Inoue M, Morita S, Watanabe Y (2010). Inner segment/outer segment junction assessed by spectral-domain optical coherence tomography in patients with idiopathic epiretinal membrane. *American Journal of Ophthalmology*.

[27] Maheshwary AS, Oster SF, Yuson RMS, Cheng L, Mojana F, Freeman WR (2010). The association between percent disruption of the photoreceptor inner segment-outer segment junction and visual acuity in diabetic macular edema. *American Journal of Ophthalmology*.

[28] Okamoto F, Sugiura Y, Okamoto Y, Hiraoka T, Oshika T (2012). Associations between metamorphopsia and foveal microstructure in patients with epiretinal membrane. *Investigative Ophthalmology and Visual Science*.

[29] Inoue M, Morita S, Watanabe Y (2011). Preoperative inner segment/outer segment junction in spectral-domain optical coherence tomography as a prognostic factor in epiretinal membrane surgery. *Retina*.

[30] Harada C, Mitamura Y, Harada T (2006). The role of cytokines and trophic factors in epiretinal membranes: involvement of signal transduction in glial cells. *Progress in Retinal and Eye Research*.

[31] Iannetti L, Accorinti M, Malagola R (2011). Role of the intravitreal growth factors in the pathogenesis of idiopathic epiretinal membrane. *Investigative Ophthalmology and Visual Science*.

